# Outcomes of peritoneal dialysis in elderly vs non-elderly patients: A systemic review and meta-analysis

**DOI:** 10.1371/journal.pone.0263534

**Published:** 2022-02-08

**Authors:** Chunling Jiang, Qiang Zheng

**Affiliations:** Department of nephrology, The affiliated People’s Hospital with Jiangsu University, Zhenjiang, Jiangsu, P.R.China; The University of Mississippi Medical Center, UNITED STATES

## Abstract

**Objectives:**

Several studies have compared outcomes of peritoneal dialysis (PD) between elderly and non-elderly patients but with variable results. We hereby designed this review to compare mortality, peritonitis, and technique survival between elderly and non-elderly patients on PD.

**Methods:**

PubMed, Embase, and Google Scholar were searched for studies comparing outcomes of PD between elderly and non-elderly patients. The last search date was 14^th^ July 2021.

**Results:**

Fourteen studies were included. 12 studies defined the elderly as ≥65 years of age and these were included in the meta-analysis. Pooled analysis of crude (RR: 2.45 95% CI: 1.36, 4.40 I^2^ = 97% p = 0.003) and adjusted data (HR: 2.80 95% CI: 2.45, 3.09 I^2^ = 0% p<0.00001) indicated a statistically significant increased risk of mortality amongst elderly patients as compared to non-elderly patients. Meta-analysis of four studies demonstrated a statistically significant increased risk of peritonitis in the elderly (RR: 1.56 95% CI: 1.18, 2.07 I^2^ = 76% p = 0.002). Pooled analysis demonstrated no statistically significant difference in technique survival between the two groups (RR: 0.95 95% CI: 0.86, 1.05 I^2^ = 86% p = 0.32).

**Conclusion:**

Elderly patients on PD have a significantly increased risk of mortality as compared to non-elderly patients. The risk of peritonitis is also significantly increased in older adults but the increased age has no impact on technique survival. Further studies are needed to strengthen our conclusions.

## Introduction

Chronic renal disease is a major health problem with high incidence worldwide. According to a recent study, the global incidence of chronic renal disease is around 11–13% and majority of the patients are in stage 3 disease or above [1]. With improvement in healthcare facilities, life expectancy has increased worldwide and a large number of elderly patients are being diagnosed with end-stage renal disease (ESRD), with a need for dialysis. Indeed, a survey in China has shown that there are 1.2 million patients with ESRD with a mean age of 63.5 years [2]. Peritoneal dialysis (PD) is a common modality available for home dialysis for elderly patients and has advantages like ease of procedure, stable hemodynamics, and reduced cost, but it still may not be feasible for all [3]. Older adults have several comorbidities, decreased dexterity, visual acuity, and diminished cognitive function, all of which can impact the safe initiation of PD [4]. Furthermore, there are concerns amongst clinicians that the high burden of physical and cognitive impairment associated with older age could increase the risk of technique failure and mortality [4]. In this context, a large number of elderly patients rely on the support of family caregivers for initiating PD. However, the burden and risks of initiating home-based PD may still not be acceptable to all family members and caregivers which is why patients are still referred to in-center hemodialysis rather than home-based PD [5].

Nonetheless, with an increasingly large number of ESRD patients, accommodating all elderly patients for thrice-weekly hemodialysis may not be feasible in the near future [5]. Furthermore, hemodialysis too is not without complications. Associated comorbidities like vascular diseases can lead to access-related complications in elderly patients on hemodialysis. Cardiovascular diseases can cause intra-dialytic hypotension and arrhythmias with prolonged recovery time after a haemodialytic session [6]. In this context, the use of PD in elderly patients has gradually increased and in the near future, a larger number of elderly patients would require to choose PD instead to hemodialysis. However, there still exists a perception that PD may also be associated with an increased risk of complications in this cohort [7]. To answer this clinical query, several studies in the past decade have compared outcomes of PD between elderly and non-elderly patients, but with variable results [8–12]. While some studies [8, 10] have reported no difference in the risk of peritonitis between elderly and non-elderly patients undergoing PD, others [12] have reported significantly increased risk of peritonitis amongst older adults. The results of one study [9] suggest that elderly PD patients have an increased risk of mortality as compared to their younger counterparts while another suggests no such difference [10]. Owing to such disparity in the study results, it is currently unclear if the elderly are able to sustain PD in a similar fashion as compared to younger ESRD patients. To the best of our knowledge, no study has been attempted to pool data to provide high-quality evidence on the exact difference in complications between elderly and non-elderly patients on PD. Given this lacuna in the literature, we hereby designed this review to compare mortality, peritonitis, and technique survival between elderly and non-elderly patients on PD.

## Material and methods

The methodological approach of our review was based on the guidelines of the PRISMA statement (Preferred Reporting Items for Systematic Reviews and Meta-analyses) [13]. We prospectively registered the review protocol on PROSPERO with registration no CRD42021267341.

### Literature search

We searched for relevant articles electronically on the databases of PubMed and Embase. We also employed Google scholar for searching gray literature. To reduce single reviewer bias, two authors searched the databases independent of each other. The search range was from the time of inception of databases up to 14^th^ July 2021. No language restriction was applied. We used the following search string for the literature search in all databases: "aged" OR “elderly” OR "older", AND "peritoneal dialysis". After the initial search, the results were deduplicated and the remaining articles were assessed by their titles and abstracts. We identified studies relevant to the review and extracted their full texts. There were evaluated by the two reviewers independent of each other for final inclusion in the review. Any discrepancies in study selection were resolved by consensus. In the end, manual scoping of the reference list of included studies was carried out for any missed references.

### Eligibility criteria

The inclusion criteria based on PICOS (population, intervention, comparison, outcomes, and study type) were formulated as follows: 1) Studies on patients undergoing PD (*Population*). We only considered studies defining elderly patients as ≥65, ≥70, or ≥ 75 years of age. 2) Studies were to compare elderly patients (*Intervention*) with non-elderly patients (*Comparison*) 3) Studies were to report either rates of mortality, peritonitis, or death-censored technique survival (*Outcomes*) 4) Studies were prospective or retrospective cohort in nature.

We excluded the following studies: 1) Studies using any other definition of elderly patients 2) Studies comparing elderly patients with a particular subset of non-elderly patients (e.g. only age group of 40–60 years) 3) Studies not reporting any of the relevant outcomes 4) Abstracts, case reports, and review articles 5) Studies reporting duplicate data.

### Data extraction and quality assessment

Data from each study was sourced by two authors independently. We extracted details of the first author, publication year, study type, study location, the definition of elderly, sample size, demographic details, body mass index, comorbidities (diabetes mellitus or cardiovascular disease), hemoglobin, serum albumin, serum creatinine, glomerular filtration rate, follow-up duration, and study outcomes. Any disagreements were resolved by discussion.

The methodological quality of studies was assessed using the Newcastle-Ottawa scale (NOS) [14]. It was conducted by two authors independently with any disagreements being resolved by discussion. Studies were assessed for selection of study population, comparability, and outcomes, with each domain being awarded a maximum of four, two, and three points respectively. The maximum score which could be awarded was nine. Studies with nine points were considered to have a low risk of bias, seven to eight points were considered to have a moderate risk of bias and those with scores of six and below possessed a high risk of bias.

### Statistical analysis

We conducted the meta-analysis using “Review Manager” (RevMan, version 5.3; Nordic Cochrane Centre [Cochrane Collaboration], Copenhagen, Denmark; 2014). All outcomes were pooled using risk ratios (RR) and 95% confidence intervals (CI). We preferred the random-effects model for the meta-analysis. We also extracted multivariable-adjusted hazard ratios (HR) of mortality when available and pooled them using the generic inverse variance function of the meta-analysis software.

Heterogeneity was assessed using the Higgins I^2^ statistic. I^2^ values of 25–50% represented low, values of 50–75% medium, and more than 75% represented substantial heterogeneity. Funnel plots were not used to assess publication bias as less than ten studies were available for each meta-analysis. A sensitivity analysis was carried out to assess the contribution of each study to the pooled estimate by removing one study one at a time and recalculating the pooled effect estimates for the remaining studies. P values <0.05 were considered statistically significant.

## Results

**Fig 1:**
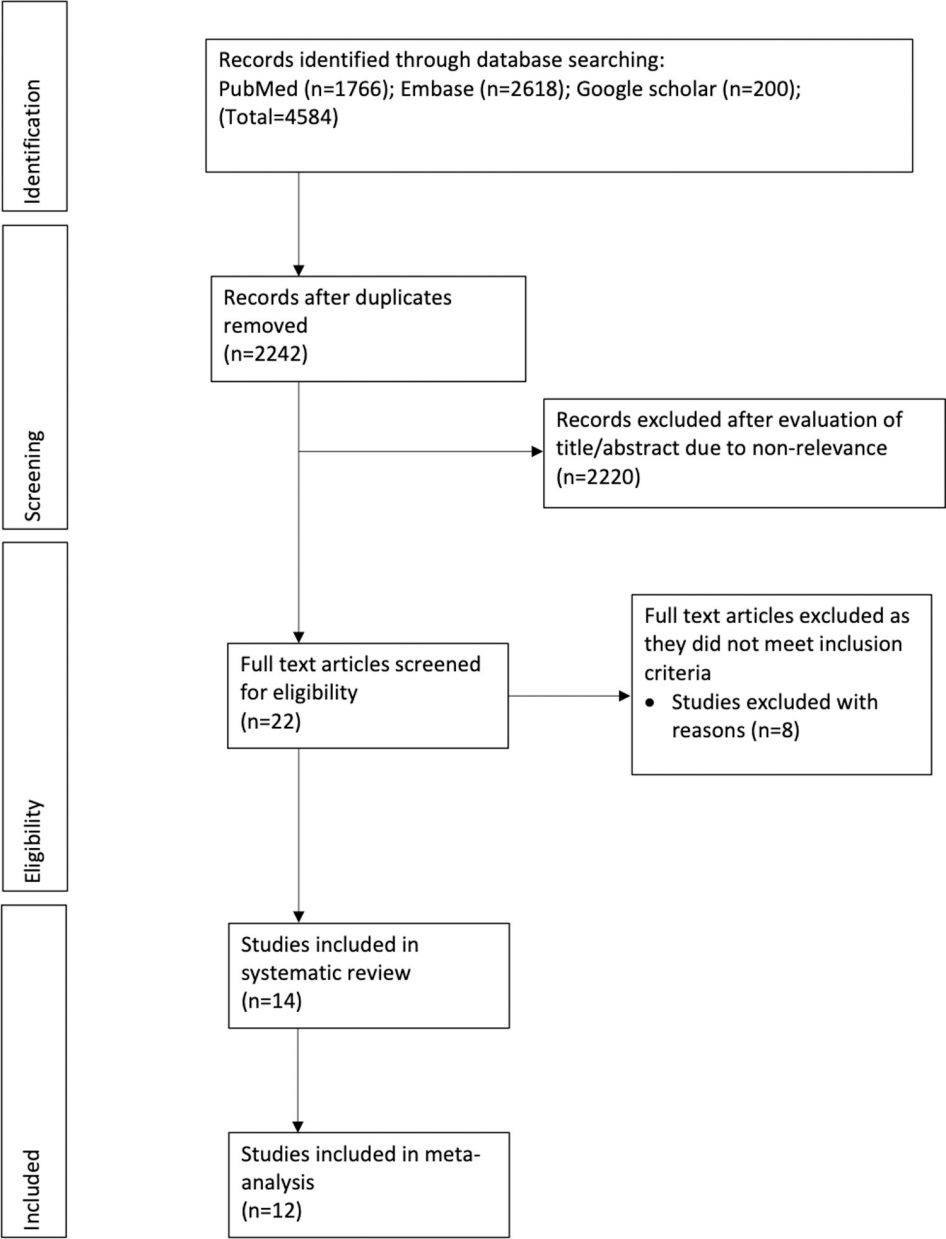
Study flow-chart.

The results of the search strategy and the number of records at each stage are presented in Fig 1. Of the 2242 unique records identified, 2220 were excluded based on title and abstract information. Twenty-two articles were analyzed by their full texts. Of these, 8 studies were excluded (Table 1) [15–22]. Finally, 14 articles were included in our systematic review and meta-analysis [8–12, 23–31].

**Table 1:** Reasons for excluded studies.

Study	Reason
Castrale 2009 [15]	No control group
Nessim 2009 [16]	Not reporting outcomes for ≥70 vs <70 age groups
Hung 2009 [18]	No control group
De Vecchi 1998 [20]	Control group restricted to 40–60 years
Taveras 2012 [22]	Data of control group not clearly presented
Smyth 2012 [21]	Only patients above 50 years of age included in the study
Wang 2015 [17]	Not comparing elderly with non-elderly patients
Jeloka 2016 [19]	No control group <65 years of age

Details of included studies are presented in Table 2. All were retrospective cohort studies published between 2000 to 2021, except for one study which was prospective in nature [31]. Eight studies were from in Asian countries, four from Europe, one from South America, and one from Australia and New Zealand. The majority of studies defined elderly patients as ≥65 years of age except for Sakai et al [8] and Duquennoy et al [12] wherein the elderly were defined as ≥70 and ≥75 years of age respectively. The sample size of the elderly group in the included studies ranged from 19 to 5176 patients while that of the non-elderly group ranged from 22 to 5223 patients. There was wide variability in the follow-up time of the included studies. The NOS scores of the included studies ranged from 6 to 8.

**Table 2:** Details of included studies.

Study	Location	Groups	Sample size	Mean/ Median age (Years)	Male gender (%)	BMI (Kg/m^2^)	DM (%)	CVD (%)	Hb (g/dL)	Serum Albumin (g/dL)	Serum creatinine (mg/dL)	GFR (mL/min/1.73 m^2^)	Follow-up	NOS
Portoles 2021 [23]	Spain	>65	777	72.9	67.1	NR	29.5	34.5	NR	NR	NR	NR	Mean 2.1 years	8
		≤65	1658	47.9	63.3		17.2	14						
Wu 2020 [9]	China	≥65	334	70.9	51.5	21.8± 3.1	53.9	68.6	9.99± 1.9	3.41± 0.5	6.68± 2.4	3.85± 2.8	Median 2.64 years	8
		<65	1619	42.4	61.3	21.6± 3.1	17.7	33.6	10.39± 2	3.75± 0.5	8.75± 2.9	4.07± 2.7		
Sakai 2018 [8]	Japan	≥70	19	77.9	68.4	21.5± 3	31.6	NR	9.9± 0.3	3.2± 0.5	8.1± 0.6	5.6± 2.1	Up to 14 years	6
		<70	109	52.1	71.5	23.4± 3.9	34.9		9.4± 0.1	3.3± 0.6	9.4± 0.2	5.7± 2.2		
Lai 2018 [10]	Italy	≥65	29	74.1	NR	26.1± 3.5	21	31	10.9± 1.6	4.08± 0.3	6.1± 2.3	NR	2 years	6
		<65	22	48.7		23.5± 3.8	9	13.6	11.12± 1.7	4.05± 0.6	6.4± 1.6			
Htay 2018 [11]	Singapore	≥65	79	73^	44	24.6± 3.8	66	52	NR	2.87± 0.6	NR	NR	NR	7
		<65	168	54	53	24.5± 4.7	46	44		3.09± 6				
Duquennoy 2016 [12]	France	≥75	3173	82^	56	NR	35	NR	NR	NR	NR	NR	Up to 10 years	8
		<75	5223	59	56		31							
Kim 2015 [24]	Korea	≥65	95	70.3	66	22± 3	NR	67	9.2± 1.4	3.3± 0.6	NR	8.9± 3.9	Up to 5 years	8
		<65	397	47.3	78.8	23± 3.5		23.9	9.2± 1.7	3.45± 0.7		7.4± 9.1		
Joshi 2014 [26]	China	≥65	148	71.3	50.7	22± 3.1	NR	53.4	9.81± 2.2	3.52± 5.1	6.3± 2.2	10± 4.6	2–6 years	8
		<65	657	43.1	58.4	21.3± 3		26	10± 2.2	3.78± 5.1	7.8± 2.8	8.97± 4		
Hiramatsu 2012 [27]	Japan	≥65	148	78.2	49.3	NR	NR	NR	9.09± 1.7	3.03± 0.7	6.35± 2.5	NR	Up to 4 years	6
		<65	99	52	65				9.4± 1.7	3.4± 0.6	8± 3			
Lim 2011 [25]	Australia & New Zealand	≥65	5176	NR	57	NR	34	55	NR	NR	NR	NR	Up to 14 years	8
		<65*	3370		50		33	15						
Yang 2007 [28]	China	≥65	145	74	57.9	NR	63.4	63.4	9.7± 1.6	3.75± 5.7	NR	NR	Up to 10 years	8
		<65	213	46.9	49.3		40.4	19.2	9.48± 1.6	3.91± 5.2				
Li 2007 [29]	Hong Kong	≥65	121	71.3	55.3	NR	54.5	NR	NR	3.09± 4.7	NR	3.11± 2.1	Up to 5 years	6
		<65	207	49.5	49.7		41.5			3.29± 5.3		3.4± 2.4		
Fernandez 2004 [30]	Chile	≥65	36	74	64	NR	31	83	NR	NR	NR	NR	Up to 4 years	6
		<65	84	55	46		13	14						
Pérez–Contreras 2000 [31]	Spain	≥65	138	72.3	54	NR	15.2	NR	NR	NR	NR	NR	NR	6
		<65	243	46	59		21.4							

*included only <50 years ^Median values.

BMI, Body mass index; DM, Diabetes mellitus; CVD, Cardiovascular disease; Hb, Hemoglobin; GFR, Glomerular filtration rate; NOS, Newcastle Ottawa scale; NR, Not reported.

### Meta-analysis

#### Mortality

**Fig 2:**
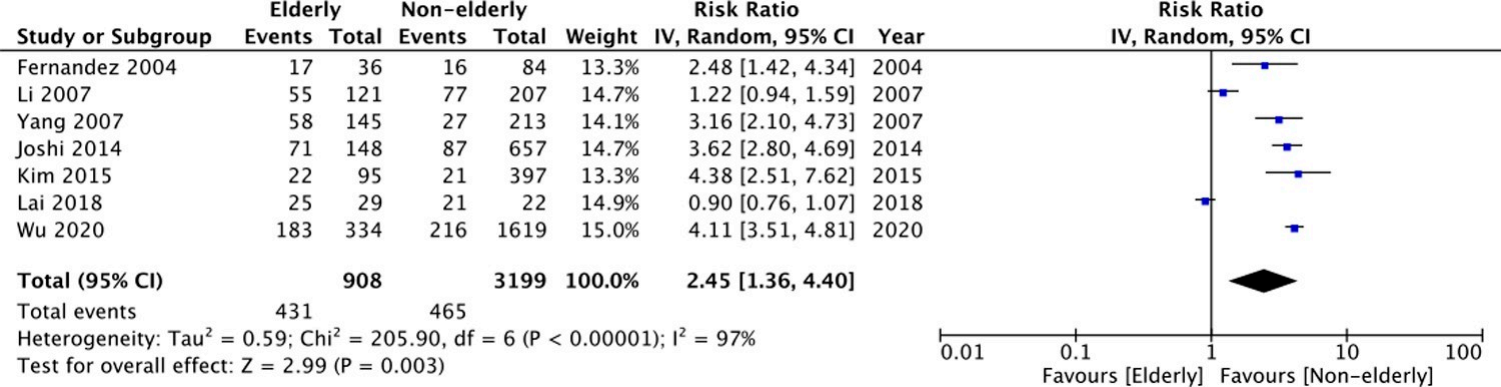
Meta-analysis of crude mortality rates between elderly vs non-elderly patients.

**Fig 3:**
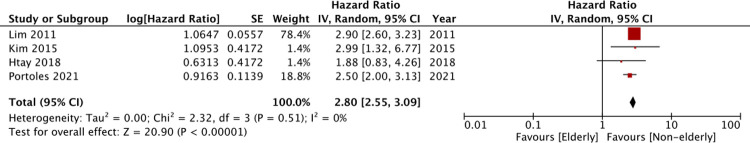
Meta-analysis of adjusted mortality rates between elderly vs non-elderly patients.

To maintain homogeneity of our meta-analysis, we included only those studies defining the elderly as ≥65 years. Of the 12 studies available for quantitative analysis, seven studies reported data on crude mortality rates. On meta-analysis, there was a statistically significant increased risk of mortality amongst elderly patients as compared to non-elderly patients undergoing PD (RR: 2.45 95% CI: 1.36, 4.40 I^2^ = 97% p = 0.003) (Fig 2). Four studies reported multivariable-adjusted HRs of mortality. On pooled analysis, the results still indicated a statistically significant increased risk of mortality amongst elderly patients (HR: 2.80 95% CI: 2.55, 3.09 I^2^ = 0% p<0.00001) (Fig 3). There was no change in the significance of either results on exclusion of any study during sensitivity analysis.

#### Peritonitis

**Fig 4:**
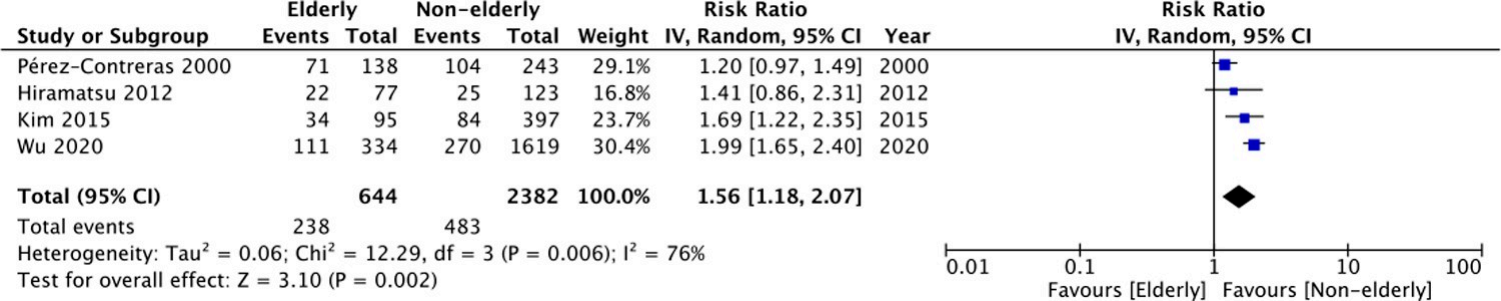
Meta-analysis of peritonitis rates between elderly vs non-elderly patients.

The method of reporting peritonitis rate was quite variable amongst the included studies. Only four studies presented data on the number of patients diagnosed with peritonitis during the follow-up period. Meta-analysis of these studies demonstrated a statistically significant increased risk of peritonitis in the elderly (RR: 1.56 95% CI: 1.18, 2.07 I^2^ = 76% p = 0.002) (Fig 4). The results were stable on sensitivity analysis. Results of the remaining studies analyzing peritonitis as one of the outcomes are presented in Table 3.

**Table 3:** Descriptive analysis of peritonitis data from the included studies.

Study	Outcome	Results
Portoles 2021 [23]	Peritonitis rate	Significantly higher in ≥65 age group vs <65 age group (0.65 vs 0.45 episodes/patient /year)
Wu 2020 [9]	Peritonitis rate	Significantly higher in ≥65 age group vs <65 age group (0.203 vs 0.145 episodes/patient /year)
	Peritonitis related mortality	Significantly higher in ≥65 age group vs <65 age group (OR: 3.57, 95% CI: 1.38–9.28)
Sakai 2018 [8]	Peritonitis rate	No significant difference between ≥70 vs <70 age groups (21.1% vs 39.4%)
Lai 2018 [10]	Peritonitis index	No significant difference between ≥65 vs <65 age group (0.03± 0.002 vs 0.03±0.001 episodes/month)
Duquennoy 2016 [12]	Incidence rate ratio(IRR) of peritonitis	Significantly lower in <75 age group as compared to ≥75 age group (IRR: 0.86, 95% CI: 0.81–0.91)
Lim 2011 [25]	Time to first peritonitis	Significantly faster in ≥65 vs <65 age group (HR: 1.11, 95% CI: 1.04–1.18)
Li 2007 [29]	12-month peritonitis free period	No difference between ≥65 vs <65 age groups (76.6% vs 76.5%)
Fernandez 2004 [30]	Peritonitis rate	No difference between ≥65 vs <65 age groups (0.16 vs 0.14 episodes/patient /year)

OR, Odds ratio; HR, Hazard ratio; CI, confidence intervals.

#### Technique survival

**Fig 5:**
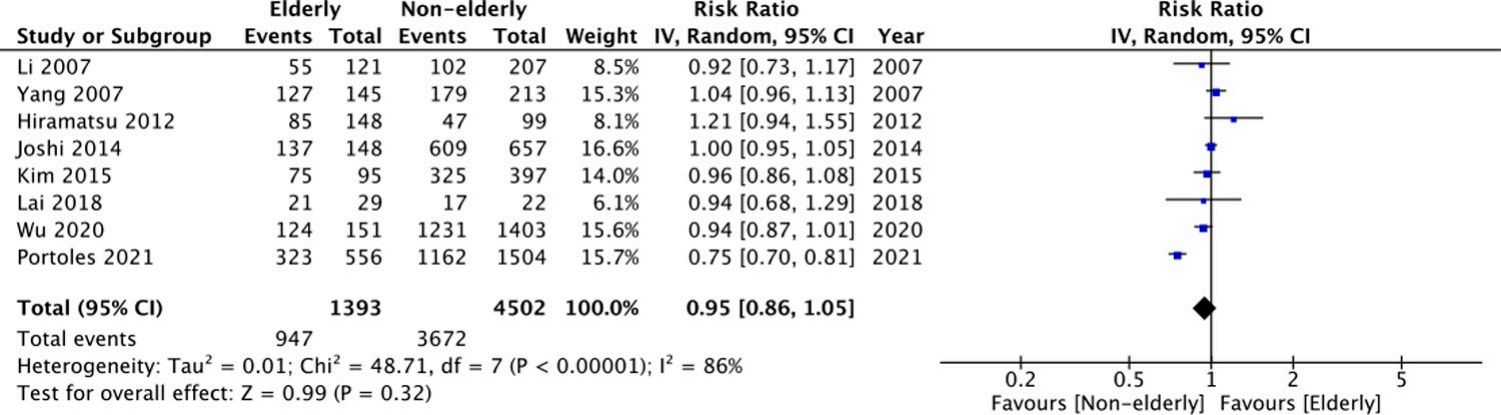
Meta-analysis of technique survival between elderly vs non-elderly patients.

Data on death-censored technique survival was reported by eight studies. Pooled analysis demonstrated no statistically significant difference between the two groups (RR: 0.95 95% CI: 0.86, 1.05 I^2^ = 86% p = 0.32) (Fig 5). On sensitivity analysis, there was no change in significance of the results on exclusion of any study.

## Discussion

Research suggests that around 50% of patients starting on dialysis are older adults. While dialysis improves patient survival, elderly patients are a low priority for kidney transplantation programs. As a result, the number of aged patients on dialysis are on the rise [32]. However, trends suggest that the choice of dialysis modality amongst the elderly is similar to that of the general population albeit with further diminished use of PD. According to the 2020 United States Renal Data System annual report, the distribution of dialysis modality amongst the elderly (>65 years) is heavily skewed towards in-center hemodialysis (87.6%) as compared to PD (9.9%). On the other hand, similar figures for the age group 18–44 years were 77.9% and 16% respectively [33]. The reduced use of PD in elderly patients is attributable to the higher number of comorbidities, functional decline, and psychosocial issues with self-care PD. Higher risk of complications in the elderly is also of particular concern which has restricted the use of PD [4]. However, with variable results amongst previous studies, this risk of increased complications has never been quantified by a meta-analysis.

In this first systematic review, we noted a statistically significant 2.45 times increased risk of mortality amongst elderly patients undergoing PD. Even pooled analysis of adjusted data, albeit with a limited number of studies, demonstrated similarly increased mortality in the ≥65 age group. Such heightened risk of death is expected due to the higher number of comorbidities and overall frailty in the elderly [34]. Older adults are also frequently malnourished with low albumin and phosphorus levels which contributes to this increased risk [24]. The increased risk was seen amongst all studies included in the quantitative analysis for both crude and adjusted data. The lone study of Lai et al [10] could not report any statistically significant difference probably due to the limited sample size of the cohort. Research has indicated that elderly patients on hemodialysis too have an increased risk of death as compared to younger patients [34]. The difference in the risk of mortality in the elderly with the two dialysis modalities is, however, questionable. In a 12-month prospective trial conducted in the UK, Lamping et al [35] did not find any difference in patients’ survival and quality of life in elderly patients on hemodialysis and PD. On the other hand, a meta-analysis by Han et al [36] have demonstrated a small increased risk of mortality with hemodialysis vs PD in the elderly (HR: 1.10 95% CI, 1.01 to 1.20).

Amongst the causes of increased mortality, Wu et al [9] have demonstrated that peritonitis-related mortality is significantly increased in the elderly as compared to younger patients. In our quantitative analysis, we noted a statistically significant 56% increased risk of peritonitis episodes in the ≥65 age group but with data from only four studies. Qualitative analysis of the remaining studies indicated contrasting results with three studies indicating an increased rate of peritonitis [9, 12, 23] while the other three demonstrating no such difference [10, 29, 30]. Such variability of the results could be related to the sample size of these studies. While the three studies reporting increased risk of peritonitis were all of large sample size, two of the studies indicating no statistically significant difference included <50 patients per arm [10, 30]. The higher rates of peritonitis in the elderly can be attributed to several factors. Foremost, neurofunctional impairment with older age with reduced visual acuity, tremors, and dementia can be an obstacle for PD [4]. Malnutrition and immune deficiency could also contribute to this heightened risk. A study by Wu et al [37] have shown that hypoalbuminemia which is an indicator for malnutrition independently leads to 1.5 times increased risk of peritonitis. To overcome the physical difficulties and functional problems, the use of assisted PD has been recommended especially for the elderly [38]. While some studies have demonstrated that the risk of peritonitis does not differ between self-care and assisted PD [39, 40], it is important to differentiate between the type of assistance. Assistance by family members or professional medical staff could be better than untrained helpers which in turn would result in reduced risk of infection [9].

Studies have shown that peritonitis is one of the most important causes for technique failure with PD [41, 42]. Despite the heightened risk of peritonitis, death-censored technique survival was not found to be significantly different between elderly and non-elderly patients in our meta-analysis. The overall risk ratio was 0.95 with a narrow 95% CI ranging from 0.86 to 1.05. This demonstrates that peritonitis episodes in the elderly can be managed similar to those of younger patients and do not necessitate discontinuation of PD and transfer to HD. Htay et al [11] in their study have demonstrated that the odds of treating peritonitis with antibiotics are similar between elderly and non-elderly patients. Furthermore, the rate of peritonitis-related catheter removal was found to be lower in the elderly and there was no difference in relapsing peritonitis or peritonitis-related hospitalization based on age group. Joshi et al [26] have suggested that the lack of difference in technique survival between elderly and non-elderly could also be related to the assistance received by elderly patients while performing PD.

Our study has some limitations. Firstly, the majority of our studies were retrospective in nature and such studies are known to have an inherent bias. Importantly selection bias with retrospective analysis can skew the study results. Secondly, the overall number of studies included in the meta-analysis was not very high. For an important outcome like the risk of peritonitis, only four studies could be quantitatively analyzed. Thirdly, the results of our study should be interpreted with caution due to the high heterogeneity seen in the meta-analyses. We were unable to explore the source of heterogeneity as all the results were stable on sensitivity analyses. However, since the included studies were performed in different setups worldwide and at different periods, we believe, several factors like baseline patient variables, variations in the dialysis protocol, the type of assistance, etc could have influenced the study results. These confounders could have contributed to the high heterogeneity in our meta-analyses.

Nevertheless, our study also has some strengths. It is the first meta-analysis to compare outcomes of elderly and non-elderly patients on PD. A detailed literature search was carried out to compare outcomes of elderly and non-elderly patients undergoing PD without any restrictions on the definition of elderly, so as to include maximum studies in the review. Additionally, in order to avoid bias representation of results, we pooled data of both crude as well as adjusted mortality rates. The stability of the results on sensitivity analysis lends some credibility to our conclusions. Lastly, a detailed qualitative description of peritonitis outcome was also carried out to provide comprehensive evidence.

Our review improves the current medical literature by providing up-to-date pooled evidence on the outcomes of elderly undergoing PD vis-à-vis younger patients. The results may have important clinical implications as they suggest higher mortality and peritonitis rates in older adults undergoing PD. Clinicians should clearly discuss the prognosis of PD in elderly patients before starting with the treatment. Also, there is a need for further research to explore the causes of higher mortality and peritonitis rates with PD in older patients.

Considering the limitations of the current studies, future studies should be prospective in nature, include a larger sample size and conduct baseline matching of patient data by propensity score analysis to provide unbiased results. Also, research should focus on the outcomes of PD in different elderly subgroups (i.e. >65 years, >75 years and >80 years) to better elucidate the outcomes of PD in the elderly. Studies should also report data on peritonitis on a common and compliable scale to assess evidence on this important outcome.

## Conclusions

To conclude, our results indicate that elderly patients on PD have a significantly increased risk of mortality as compared to the non-elderly patients. The risk of peritonitis is also significantly increased in older adults but the increased age has no impact on technique survival. However, considering the limitations of the current review, further studies are needed to strengthen our conclusions.
